# Zinc as an Adjuvant Therapy in Acute Severe Pneumonia in Children Aged Two Months to Two Years: A Double-Blind Randomized Controlled Trial

**DOI:** 10.7759/cureus.82402

**Published:** 2025-04-16

**Authors:** SK Padmini, Kasa Mahesh, H Durgappa, Sathiyanarayanan Sathiyamoorthi

**Affiliations:** 1 Pediatrics, Dr. Pinnamaneni Siddhartha Institute of Medical Sciences and Research Foundation, Chinna Avutapalli, IND; 2 Pediatrics, Ballari Medical College and Research Centre, Ballari, IND; 3 Community and Family Medicine, All India Institute of Medical Sciences, Mangalagiri, Mangalagiri, IND

**Keywords:** nutritional deficiencies, pneumonia, randomized controlled trial, severe pneumonia, zinc supplementation

## Abstract

Introduction: The study aimed to evaluate the efficacy of zinc supplementation, in addition to standard antimicrobial therapy, in the treatment of severe pneumonia among hospitalized rural Indian children aged 2-24 months. It also sought to identify factors associated with the development of pneumonia in this population.

Methodology: A hospital-based, randomized, double-blinded, placebo-controlled trial was conducted from September to December 2022, registered with the Clinical Trial Registry of India (CTRI/2022/09/046070). A total of 94 children with severe pneumonia were randomly assigned to receive either 10 mg of zinc gluconate (2.5 ml) or a placebo twice daily during hospitalization, along with standard therapy. Clinical signs, symptoms, and laboratory parameters were monitored, and baseline characteristics were compared between the groups.

Results: The median duration of cough and cold in the Zinc group was significantly shorter compared to the Control group (p = 0.013, p = 0.005, respectively). Among stunted children, those in the Zinc group required about 3.12 fewer days for recovery compared to those in the Control group, and this difference was statistically significant (p = 0.05). However, no statistically significant differences were observed between the groups in the duration of tachypnea, hypoxia, chest indrawing, inability to feed, lethargy, severe illness, or overall hospitalization.

Conclusion: While zinc supplementation demonstrated potential benefits such as reduced hospital stay and faster symptom resolution, these differences were not statistically significant. Further research is needed to validate zinc role in improving clinical outcomes in severe pediatric pneumonia.

## Introduction

Pneumonia is the leading cause of pediatric morbidity and mortality worldwide, with an estimated eight million deaths occurring annually in children under five years of age [1]. Pneumonia contributes to approximately 20% of these deaths, while diarrhea accounts for about 25% [2]. The incidence of pneumonia is 10 times higher, and the mortality rate nearly 2,000 times higher in developing countries compared to developed ones [3]. In India, which bears the highest global burden of pneumonia, an estimated 370,000 children die of the disease each year [4]. Early diagnosis and prompt empiric antibiotic therapy have reduced pneumonia-related deaths by 47% [5]. However, the efficacy of case management strategies is significantly influenced by poor nutritional status [6,7]. Undernutrition is associated with more severe pneumonia, frequent complications, prolonged infection episodes, and higher case fatality rates [8].

In India, deficiencies in iron, zinc, and vitamins A and D are common among young children and are linked to an increased risk of developing pneumonia [9]. Zinc, an essential nutrient, is the most deficient during weaning, and its deficiency is associated with 4.4% of deaths and 3.8% of lost disability-adjusted life years (DALYs) in children aged six to 59 months across Africa, Latin America, and Asia [10,11]. Zinc deficiency weakens the immune system, leading to increased susceptibility to infections, including pneumonia and gastroenteritis [12]. Although zinc is recommended by the World Health Organization (WHO) for diarrhea treatment [13,14], its role in pneumonia treatment remains unclear. While a clinical trial in Bangladesh showed benefits [15], studies in India [16] and Australia [17] did not. This study was undertaken to evaluate the efficacy of zinc supplementation as an adjunct therapy in acute severe pneumonia. 

Objective

To study the efficacy of zinc supplementation as an adjunct therapy to acute severe pneumonia in hospitalized children aged two months to two years receiving standard antibiotic treatment.

## Materials and methods

Study design

A hospital-based, randomized, double-blinded, placebo-controlled trial was conducted to assess the efficacy of zinc supplementation as adjunct therapy in children with acute severe pneumonia hospitalized in the pediatrics department of a tertiary care hospital between September 2022 and December 2022.

Inclusion criteria

Children aged two months to two years diagnosed with severe pneumonia and admitted to the hospital were included in the study after obtaining written informed consent from at least one parent. Severe pneumonia was defined as a respiratory rate of 50/min or higher, accompanied by crepitations on auscultation and the presence of any one of the following danger signs: lethargy, inability to feed, chest indrawing, or central cyanosis. The cause of lower respiratory infections (LRIs) caused by a virus or bacteria (*Haemophilus influenzae* type b or *S. pneumoniae*) is difficult to determine because current techniques to establish bacterial etiology lack sensitivity and specificity. The results of pharyngeal cultures do not always reveal the pathogen that is the cause of the LRI. Bacterial cultures of lung aspirate specimens are often considered the gold standard, but they are not practical in resource-constrained settings; the empirical management is similar for LRIs. Hence, all children with severe pneumonia (viral/bacterial) were included in the study.

Exclusion criteria included children with chronic cardiac or renal disease, any illness requiring ventilation, severe malnutrition needing immediate nutritional rehabilitation, current zinc supplementation, any illness requiring hospitalization in the past 21 days, or those with a known allergy to zinc or its components.

Variables

The following data variables were collected at enrollment: sociodemographic data, age, sex, region, religion, socioeconomic status, anthropometric data like weight, height, mid-arm circumference, blood parameters like complete blood count, C-reactive protein, blood urea, serum creatinine, serum electrolytes, chest X-ray, urine routine and baseline serum zinc levels by atomic absorption spectroscopy method. Clinical assessments, such as chest indrawing, respiratory rate, nasal flaring, fever, and cyanosis, were also done. 

Sample size calculation

The sample size was calculated based on the primary outcome, "duration of hospitalization." A pilot study conducted at the institute provided the mean (9.75) and standard deviation (3.29). Considering a 5% chance of Type I error, 20% chance of Type II error, 10% loss to follow-up, and a minimal clinically relevant difference of 2 days, the sample size calculation formula yielded a requirement of 47 subjects per group.

Recruitment

**Figure 1:**
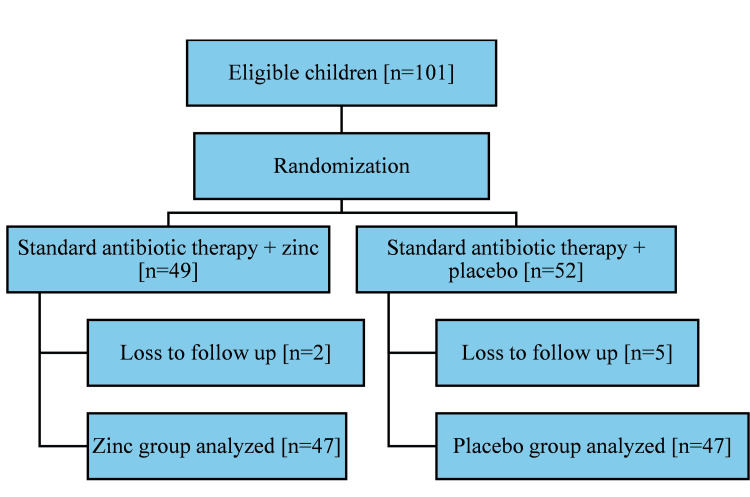
Study flow diagram

Children diagnosed with severe pneumonia, meeting the inclusion criteria, were enrolled in the study after obtaining written informed consent from their parents (Figure 1).

Randomization, blinding, and treatment allocation

Following baseline data collection, children were randomly assigned to receive either adjunctive zinc therapy or a placebo. Randomization was achieved through a computer-generated list using permuted blocks of 20, prepared by an independent researcher. The participants were assigned to either the intervention group (zinc) or the placebo group based on the next consecutive regimen number in the list. Care providers and study team members were blinded to the allocation. The trial was registered with the Clinical Trial Registry of India (CTRI/2022/09/046070). 

To ensure blinding, both the zinc syrup and the placebo (sterile distilled water) were dispensed in identical bottles with similar labeling and packaging. Although water does not have the same taste or viscosity as zinc syrup, the administration process and appearance of the bottles ensured that neither participants, caregivers, nor investigators could distinguish between the two.

Intervention Procedure

All the children received standard antibiotic treatment and were randomized to the zinc or placebo group. In the zinc group, 10 mg elemental zinc (zinc gluconate) in 2.5 mL syrup was administered twice a day for seven days, and the placebo group was administered 2.5 mL sterile distilled water. The medication was administered at noon and midnight to prevent gastrointestinal side effects.

Data Collection

Data were collected through face-to-face interviews with the mother and clinical examination of the child. Data on sociodemographic, anthropometric, blood parameters, and clinical profile were recorded at enrollment. All clinical parameters, including respiratory rate, chest indrawing, fever, oxygen saturation (SpO2), and other signs, were monitored and recorded daily during the hospital stay.

Outcome Variables

Primary outcome measures included the duration of hospitalization, duration of severe pneumonia, pneumonia, nil-per-oral status, intravenous fluids, oxygen use, and treatment failure requiring second- or third-line antibiotics. Clinical parameters such as days of illness, antibiotic use prior to admission, respiratory rate, chest indrawing, nasal flaring, fever, cough, wheeze, lethargy, cyanosis, and need for nil-per-oral, IV fluids, and oxygen were monitored throughout the hospitalization period.

Statistical analysis

Data was analyzed using the Statistical Package for the Social Sciences version 16.0 (SPSS Inc., Chicago, IL). Descriptive statistics were presented as frequencies and proportions. Continuous variables were expressed as mean and standard deviation or median and interquartile range as appropriate. Associations between categorical variables were assessed using chi-square or Fisher’s exact test, while associations between continuous variables were analyzed using the independent t-test. Paired sample t-tests were used to analyze quantitative data within the same group. A p-value of <0.05 was considered statistically significant.

Ethical considerations

The study was conducted in accordance with ethical guidelines for clinical research. Ethical approval was obtained from the institutional ethical committee, ensuring that the rights and welfare of participants were protected. Written informed consent was obtained for participation in the study and use of the patient data for research and educational purposes. The procedures follow the guidelines laid down in the Declaration of Helsinki 2008.

## Results

The study included 94 children (47 in each group) diagnosed with severe pneumonia. The mean age (± SD) in months for the Control group was 12.0 (± 6.74), while it was 11.3 (± 6.14) in the Zinc group. The minimum and maximum ages in both groups ranged from two months to 24 months. The baseline characteristics of both groups at the time of admission were compared using the chi-square test, and no statistically significant differences were found (Table 1).

**Table 1:** Baseline characteristics among the study subjects at the time of admission (n=94) The chi-square test was used for analysis. ^* ^P-value ≤ 0.05 was considered statistically significant.

Variables		Control Group		Zinc Group		P- value
Gender	Category	Frequency	Percentage	Frequency	Percentage	
	Male	23	48.9	29	61.7	0.213
	Female	24	51.1	18	38.3	
Age group	0-1 year (Infants)	30	63.8	30	63.8	1.0
	1-2 years	17	36.2	17	36.2	
Religion	Hindu	40	85.1	39	83.0	0.346
	Muslim	05	10.6	07	14.9	
	Christian	02	4.3	01	2.1	
Socioeconomic status	Middle	05	10.6	10	21.3	0.817
	Lower middle	30	63.8	25	53.2	
	Lower	12	25.6	12	25.5	
Clinical symptoms	Fever	47	100	47	100	-
	Cough	47	100	47	100	-
	Cold	47	100	46	97.9	1.00
	Hurried breathing	45	95.7	46	97.9	1.00
	Convulsions	03	6.4	04	8.5	1.00
	Nasal flaring	45	95.7	46	97.9	1.00
	Lethargy/irritability	19	44.2	24	51.1	0.301
	Cyanosis	0	0	0	0	-
	Vitamin A deficiency	07	14.9	07	14.9	1.00
	Vitamin D deficiency	08	17.0	09	19.1	0.789

In the Control group, the mean (± SD) weight was 7.3 (± 2.2), height was 69.8 (± 8.2), head circumference was 43.0 (± 2.73), and mid-arm circumference was 12.3 (± 1.31). In the Zinc group, the mean (± SD) weight was 6.9 (± 1.88), height was 67.2 (± 9.57), head circumference was 43.9 (± 6.27), and mid-arm circumference was 12.0 (± 1.50). The weight for age and height for Age were categorized according to the Indian Academy of Pediatrics (IAP) classification for malnutrition [18]. The differences in anthropometry were not statistically significant between the groups. Clinical parameters such as air entry, breathing type, intercostal retraction, crepitations, and values of blood parameters, including hemoglobin, total WBC count, differential count, C-reactive protein (CRP), urea, and creatinine, were equally distributed between the groups (Table 2).

**Table 2:** Anthropometry and laboratory parameters among the study subjects (n=94) Student’s t-test was used to analyze anthropometry, while the chi-square test was used for other variables. * P-value ≤ 0.05 was considered statistically significant.

Variable	Control Group (n = 47)	Zinc Group (n = 47)	P-value
Anthropometry			
Weight (kg) (Mean ± SD)	7.3 ± 2.2	6.9 ± 1.88	0.322
Height (cm) (Mean ± SD)	69.8 ± 8.2	67.2 ± 9.57	0.164
Head circumference (cm) (Mean ± SD)	43.0 ± 2.73	43.9 ± 6.27	0.375
Mid-arm circumference (cm) (Mean ± SD)	12.3 ± 1.31	12.0 ± 1.50	0.392
Weight for age			
Normal	28 (59.6%)	24 (51.1%)	0.331
Grade I	8 (17.0%)	6 (12.8%)	
Grade II	2 (4.3%)	6 (12.8%)	
Grade III	6 (12.8%)	10 (21.3%)	
Grade IV	3 (6.4%)	1 (2.1%)	
Height for age			
Normal	32 (68.1%)	31 (66.0%)	0.826
Stunted	15 (31.9%)	16 (34.0%)	
Hemoglobin levels (g/dL)			
Day 1	9.07	9.35	0.005*
Day 5	9.52	9.99	
Chest X-ray findings			
Bronchopneumonia	38 (80.9%)	41 (87.2%)	-
Lobar pneumonia	9 (19.1%)	6 (12.8%)	-

The baseline mean (± SD) serum zinc levels at the time of admission were 71.92 mcg/dL (± 24.61) in the Control group and 72.21 mcg/dL (± 27.70) in the Zinc group. Chest X-ray results taken on the day of admission revealed that 38 (80.9%) subjects in the Control group had features suggestive of bronchopneumonia, compared to 41 (87.2%) in the Zinc group. Additionally, nine (19.1%) subjects in the Control group had lobar pneumonia, compared to 6 (12.8%) in the Zinc group. These differences were not statistically significant.

Biochemical parameters on day five were compared with baseline values at admission, and except for mean hemoglobin levels, other parameters were comparable. The mean hemoglobin levels increased from 9.07 g/dL on day one to 9.52 g/dL on day five in the Control group, compared to an increase from 9.35 g/dL on day one to 9.99 g/dL in the Zinc group. The rise in hemoglobin levels was greater in the Zinc group compared to the Control group, and this difference was statistically significant (p = 0.005).

The effect of zinc supplementation on clinical symptoms, signs, outcomes, and improvements was compared between the two groups using Mood’s Median test. The median duration of cough and cold in the Zinc group was significantly shorter compared to the Control group (p = 0.013, 0.005, respectively). However, there were no significant improvements in other clinical outcomes with zinc supplementation (Table 3).

**Table 3:** Effect of zinc supplementation on clinical outcomes between groups Mood's Median test was used for analysis. * P-value ≤ 0.05 was considered statistically significant.

Variables		Control Group		Zinc Group		P- value
Clinical symptoms		Median (days)	IQR	Median (days)	IQR	
	Fever	5	4, 7	5	4, 6	0.677
	Cough	8	6, 10	7	5, 8	0.013*
	Cold	8	6, 10	7	5, 8	0.005*
	Hurried Breathing	4	3, 5	3	3, 4	0.315
	Convulsions	2	1.5, 2	1	1, 1.3	0.486
Clinical Signs	Tachypnoea	4	3, 5	3	3, 4	0.315
	Nasal Flaring	4	3, 5	3	3, 4	0.174
	Lethargy/ Irritability	2	1.5, 3	2	1, 2.3	0.389
Clinical improvement	No of days SpO2 < 90%	4	3, 5	3	3, 4	0.119
	No of days in Nil per oral	4	3, 4.5	3	3, 4	0.466
	No of days on IV fluids	5	4, 6	5	4, 6	0.655
	No of days on O2 support	4	3, 5	3	3, 4	0.397
Clinical outcome	Duration of stay in hospital	10	7, 13.5	7	7, 10	0.680
	No of days taken for Recovery	12	10, 18	12	10, 14.5	0.402

As malnutrition plays an important role in the treatment of ARI, outcome variables were compared among malnourished and stunted children. There were 42 malnourished children and 31 stunted children. Among stunted children, those in the Zinc group required about 3.12 fewer days for recovery compared to those in the Control group, and this difference was statistically significant (p = 0.05). No significant differences were observed in other parameters (Table 4).

**Table 4:** Effect of Zinc on Clinical Outcome (In days) among malnourished and stunted children Student’s t-test was used for analysis. ^*^ P-value ≤ 0.05 was considered statistically significant.

Clinical Outcome	Control Group (n=19)		Zinc group (n= 23)		t	P-value
	Mean	SD	Mean	SD		
Malnourished children (N=42)						
Duration of stay in hospital	11.79	3.56	10.87	3.36	-1.112	0.267
No. of days taken for recovery	16.32	5.07	14.65	4.49	-0.854	0.399
Stunted children (n=31)						
Duration of stay in hospital	12.93	3.08	11.13	3.61	-1.959	0.146
No. of days taken for recovery	17.87	4.51	14.75	4.32	-1.502	0.05*

**Figure 2:**
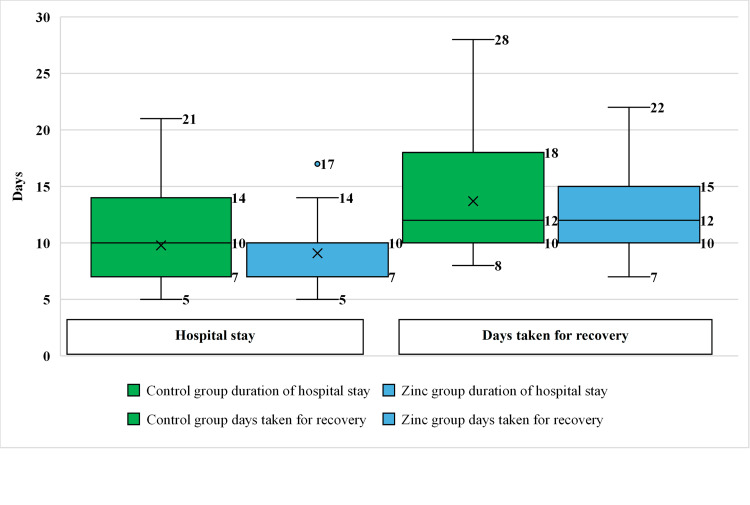
Effect of zinc supplementation on clinical outcomes among the groups (days)

The effect of zinc supplementation on clinical outcomes was studied by comparing the median duration of stay in hospital and number of days taken for recovery between groups, which was found to be statistically insignificant using Mood's Median test. The median duration of hospital stay was 10 days in the Control group and seven days in the Zinc group, whereas the median number of days for recovery was 10 in both groups (Figure 2). 

The requirement for second- or third-line antibiotics was compared between the Control and Zinc groups using the chi-square test. About 31.9% of the subjects in the Control group required second- or third-line antibiotics, compared to only 23.4% in the Zinc group. This difference was not statistically significant (p = 0.356). Similarly, the requirement for blood transfusion was compared between the two groups using the chi-square test. About 17% of the subjects in the Control group required a blood transfusion, compared to 23.4% in the Zinc group. This difference was also not statistically significant (p = 0.441).

## Discussion

In the present study, both the Control and Zinc groups consisted of 30 (61.8%) infants and 17 (36.2%) children in the age group of one to two years, which aligns with the study conducted by Bose et al. [16]. There was a balanced gender distribution in the Control group, while the Zinc group had a slightly higher number of male subjects. This finding is consistent with Shah et al. [19], who reported 62% males and 38% females in the Zinc group, compared to 68% females and 32% males in the placebo group.

The biological plausibility of zinc’s role in pneumonia recovery lies in its critical function in immune modulation, epithelial barrier integrity, and anti-inflammatory responses. Zinc enhances T-cell function, promotes mucosal immunity, and reduces oxidative stress, which may contribute to faster recovery from respiratory infections.

In a study done by Rajasekaran et al. [20], fever (100%) and cough (94%) were the predominant symptoms, followed by refusal to feed (64%) and lethargy (26%). In the present study, 100% of subjects in both the zinc and placebo groups had fever and cough. Cold was present in 100% of subjects in the placebo group and 97.9% in the zinc group; hurried breathing in 97.9% of zinc group and 95.6% of the placebo group. This shows that there was no selection bias while assigning the subjects to either the Control or the Zinc group, with respect to clinical presentation. But Wadhwa et al. [21] reported that wheezing was the most common symptom present in about 54.7% in both the zinc and placebo group, followed by fever, which was present in 33.9% inthe zinc group and 38% in the placebo group.

In terms of malnutrition, 41.3% of children in the Zinc group and 37.6% in the placebo group were malnourished in the study by Bose et al. [16], while Rajasekaran et al. [20] reported that 22% were malnourished in both groups. Similarly, 42 children in the present study were identified as malnourished, a finding consistent with Shah et al. [19].

Regarding baseline hemoglobin levels, the mean (± SD) in the placebo group was 9.07 (± 1.71), compared to 9.35 (± 1.9) in the Zinc group. This result is consistent with the findings of several other studies [16,21,22], which reported a high prevalence of anemia among the study participants. No other studies have specifically compared serial biochemical measurements with the effect of zinc supplementation, making this aspect of the present study novel. The baseline zinc levels in this study were comparable to those observed in other Indian studies [20,22].

The resolution of symptoms was observed to occur earlier in the Zinc group compared to the placebo group, with the difference in the mean duration of cough and cold being statistically significant. However, the mean duration of tachypnea, nasal flaring, and lethargy did not show a statistically significant difference between the groups, which contrasts with findings by Hashemian et al. [23], who observed a statistically significant difference. Many other studies, such as those by Bose et al. [16], Wadhwa et al. [21], and others [24], did not find a significant difference in the time required for clinical signs to resolve in the Zinc group compared to the placebo group. While the present study did show a marginal reduction in the duration of clinical improvement in the Zinc group, this difference was not statistically significant, which is in line with the results of several other studies [16,19,24].

In terms of antibiotic usage, 31.9% of subjects in the Control group required second- or third-line antibiotics, compared to 23.4% in the Zinc group. This result is consistent with findings by Wadhwa N et al. [20], who reported that 11.8% of the Zinc group and 8.4% of the placebo group required a change in antibiotics. These findings align with the results of other studies [19,25].

A meta-analysis of clinical trials evaluating the preventive role of zinc supplementation in pneumonia has shown that daily supplementation led to a 14% reduction in the risk of diarrhea and an 8% reduction in the risk of pneumonia [26]. However, the evidence supporting zinc as a beneficial therapy in pneumonia remains conflicting. These findings are summarized in Table 5.

**Table 5:** Comparison of studies on effect of zinc supplementation in treatment of acute respiratory infections

Studies	Year	Place	Age group studied	Conclusion on zinc
Hashemian et al. [23]	2021	Iran	2 to 60 months	Beneficial
Basnet et al. [27]	2018	Nepal	2 to 35 months	Beneficial
Rajagembeeran et al. [22]	2015	India	2 to 24 months	Beneficial
Qasemzadeh et al. [28]	2014	Iran	3 to 60 months	Beneficial
Mahalanabis et al. [29]	2004	India	2 to 24 months	Beneficial
Wang and Song [30]	2017	China	Meta-analysis	Beneficial
Lassi et al. [31]	2014	Canada	Systematic review	Beneficial
Sempértegui et al. [25]	2014	Ecuador	2 to 59 months	No difference
Howie et al. [24]	2018	Gambia	2 to 59 months	No difference
Srinivasan et al. [32]	2012	Uganda	6 to 59 months	No difference
Wadhwa et al. [21]	2010	India	2 to 24 months	No difference
Shah et al. [19]	2009	Nepal	2 to 60 months	No difference
Bose et al. [16]	2006	India	2 to 23 months	No difference
Fataki et al. [33]	2014	Tanzania	6 to 36 months	No difference

## Conclusions

Baseline characteristics of the two groups, such as gender, age group, SES, religion, symptoms, signs, examination findings, lab investigation values, including zinc, were comparable, and there was no statistical difference in any of the parameters. Zinc supplementation had a significant improvement in mean Hb levels compared to the placebo group. Among stunted children, zinc supplementation had a significant reduction in the number of days needed for recovery compared to the placebo group.

Zinc supplementation had an overall effect in decreasing the duration of hospital stay, the number of days needed for recovery, the number of days required to achieve clinical improvement, symptom and sign resolution, and the requirement for second- and third-line antibiotics, but these differences were not statistically significant. As conflicting evidence is available with respect to the role of zinc in the treatment of severe pneumonia, further research in representative settings is required.
